# Journey to the center of the heart: Bilateral absent superior vena cava with heart block

**DOI:** 10.21542/gcsp.2020.33

**Published:** 2020-12-31

**Authors:** Ahmed Elborae, Ramy Doss, Mahmoud Shaaban, Ahmed A. Elkhouly, Mohamed Abdullah, Khalid Sorour

**Affiliations:** 1 Faculty of Medicine, Cairo University, Egypt; 2 Aswan Heart Centre, Aswan, Egypt; 3 Faculty of Medicine, Tanta University, Egypt; 4 St. Francis Medical Center, Grand Island, NE 68803, USA

## Abstract

Bilaterally absent superior vena cava (SVC) is extremely rare anomaly with a few case reports in the literature. Without associated congenital cardiac disease, these anomalies are asymptomatic. This report describes an adult patient with bilaterally absent SVC presenting with Mobitz type II heart block and a structurally normal heart.

## Case report

**Figure 1.**
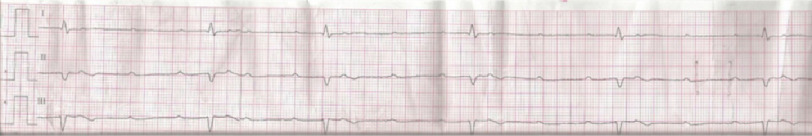
A rhythm strip ECG showing Mobitz type II heart block with 3:1 conduction.

A previously healthy 59-year-old male Egyptian patient presented to the emergency department following a syncopal attack. He also reported progressive shortness of breath and bilateral lower limb oedema over the last couple of months. On examination, his blood pressure was 180/110 mmHg with a regular pulse of 30 beats per minute. Chest examination revealed bilateral diminished air entry over the lung base with fine inspiratory crepitation. He also showed bilateral pitting lower limb oedema up to the knee. His cardiac examination revealed muffled s1 with normal s2 and no additional sounds or murmur. An electrocardiogram (ECG) was performed and revealed Mobitz type II heart block with 3:1 conduction (Figure 1).

While attempting insertion of temporary pacing wire from the right internal jugular vein (IJV) toward the superior vena cava (SVC); it would rather take a left para-median course and cross the infra-diaphragmatic border caudally.

**Figure 2.**
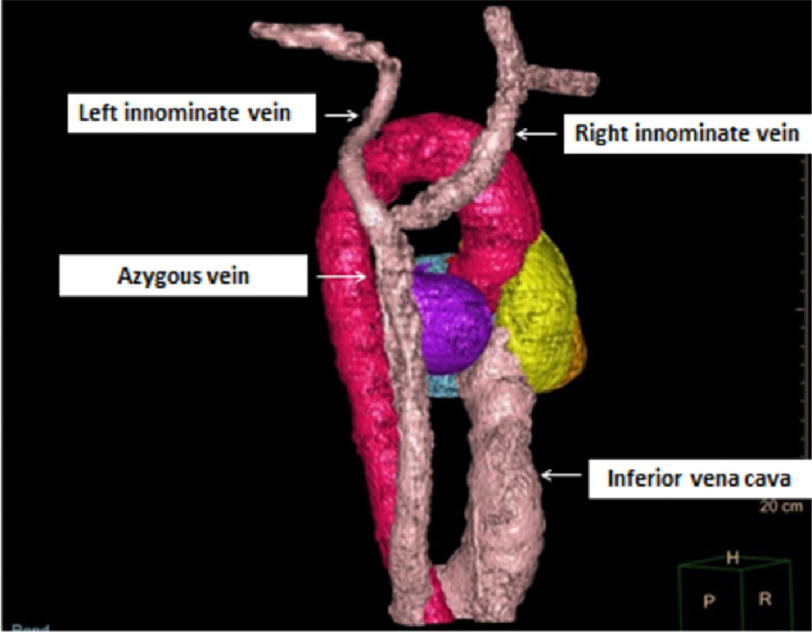
A CT chest with 3D reconstruction. It shows bilateral absence of the SVC with both innominate veins drain into the azygous vein that joins the IVC eventually. CT: Computerized tomography, SVC: superior vena cava, IVC: inferior vena cava.

**Figure 3.**
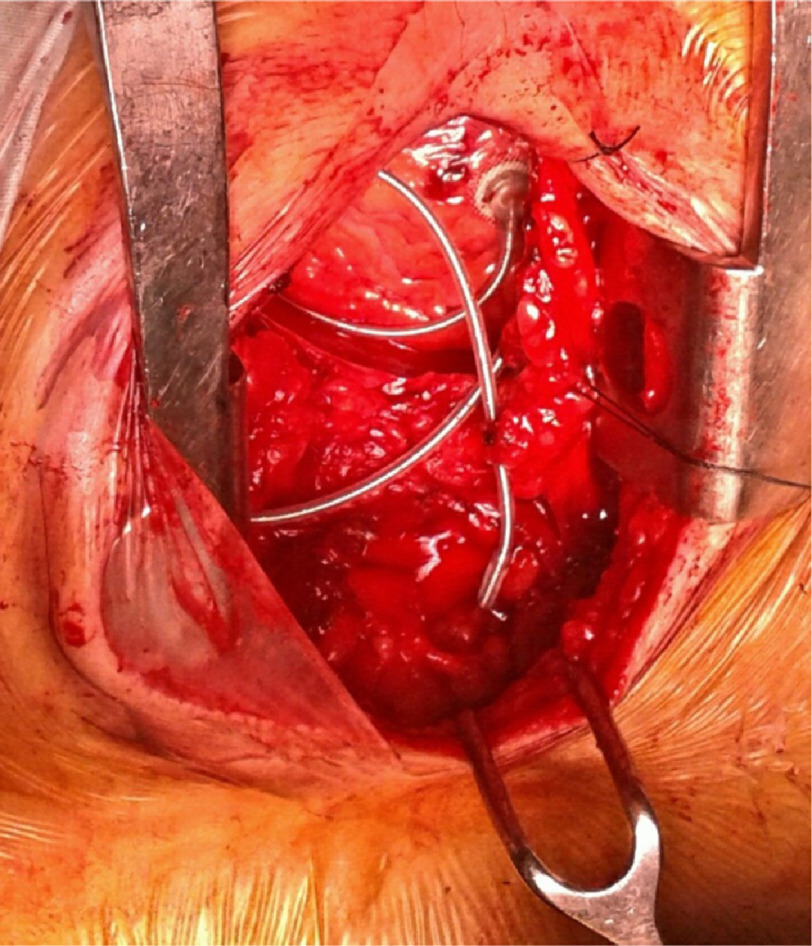
Limited sternotomy showing epicardial lead insertion.

A contrast injection into that vessel suggested that it might be a dilated azygous vein. Surprisingly, the venous flow was reversed towards the inferior caval vein (IVC). Successful temporary right ventricular pacing was easily achieved from the right femoral venous access through the IVC into the right atrium and ventricle. The patient reported immediate improvement and was transferred to the intermediate care unit for observation. There were no laboratory abnormalities that could explain this conduction defect. Trans-thoracic echocardiography revealed concentric left ventricular hypertrophy with dilated left atrium and dilated IVC. A computed tomography (CT) chest with contrast confirmed the absence of both SVC with both brachiocephalic veins draining into azygous vein to the IVC (Figure 2). Permanent pacing was achieved through epicardial leads. (Figure 3).

## Discussion

During the fourth gestational week, the right and the left anterior cardinal veins drain the upper portion of the embryo. Each anterior cardinal vein drains into a common cardinal vein before entering the embryological heart.

By the eighth gestational week, a venous anastomosis connects the two anterior cardinal veins while the left common cardinal vein completely regresses to become the ligament of Marshall. With this regression, the anastomotic vein between the two precardinal veins becomes the brachio-cephalic vein and the right precardinal vein and the right common cardinal vein form the SVC. Failure of this normal regression can lead to the formation of a persistent left SVC, the most common venous abnormality.^1^

Very rarely, bilateral regression of common cardinal veins occurs leading to the bilateral absence of the superior vena cava. The left and right head and neck veins connect to the azygos and hemiazygos veins, respectively. Then it drains into the right atrium, inferior vena cava, left renal vein, or a complex subdiaphragmatic plexus of veins.^2^

To our knowledge, only 14 cases were reported to have bilaterally absent SVC. The true prevalence of this condition is unknown but is probably much higher. Most cases were diagnosed either incidentally during chest imaging^3,4,5^ or during a complicated vascular access during pacemaker implantation for complete heart block^6,7^ or electrophysiological ablation procedure.^8^ In one case, superior vena cava syndrome was reported in an adult male with bilateral absent SVC.^9^

The association between absent superior vena cava and conduction block, as in our case, has been previously reported in two octogenarian patients.^6,7^ Such association is more likely to be a coincidence, with the absent SVC being unmasked by need for temporary pacemaker insertion. Another possibility is an associated developmental abnormality of conduction system that accelerated sclerodegenerative changes. Some embryological studies have shown that the sinus venosus myocardium is incorporated in the AV canal in the posterior region of the AVN. The ‘pacemaker field’ was shown to be embedded in the zone that also generates other cardiac-related tissues, including the vena cava.^10^ This hypothesis remains unproven given the rarity of the condition and absent reports of early-onset heart block associated with bilateral absent SVC in literature.

## Follow up

The patient is doing fine and is currently asymptomatic.

## Conclusion

Despite its rarity, the awareness about bilateral absent SVC anomaly is essential in setting of transvenous procedures as cardiac device implantation as well as some cardiac (e.g., Glenn procedure) and non-cardiac surgeries (e.g., hepatectomy), where clamping of the inferior vena cava would likely lead to death due to total venous obstruction.

## Abbreviations List

CT: Computed tomography.
ECG: Electrocardiogram.
IJV: Internal jugular vein.
IVC: Inferior vena cava.
SVC: Superior vena cava.
